# Predicting the recurrence of spontaneous intracerebral hemorrhage using a machine learning model

**DOI:** 10.3389/fneur.2024.1407014

**Published:** 2024-05-22

**Authors:** Chaohua Cui, Jiaona Lan, Zhenxian Lao, Tianyu Xia, Tonghua Long

**Affiliations:** Life Science and Clinical Medicine Research Center, Affiliated Hospital of Youjiang Medical University for Nationalities, Baise, China

**Keywords:** intracerebral hemorrhage, recurrence, predicting, model, machine learning

## Abstract

**Background:**

Recurrence can worsen conditions and increase mortality in ICH patients. Predicting the recurrence risk and preventing or treating these patients is a rational strategy to improve outcomes potentially. A machine learning model with improved performance is necessary to predict recurrence.

**Methods:**

We collected data from ICH patients in two hospitals for our retrospective training cohort and prospective testing cohort. The outcome was the recurrence within one year. We constructed logistic regression, support vector machine (SVM), decision trees, Voting Classifier, random forest, and XGBoost models for prediction.

**Results:**

The model included age, NIHSS score at discharge, hematoma volume at admission and discharge, PLT, AST, and CRP levels at admission, use of hypotensive drugs and history of stroke. In internal validation, logistic regression demonstrated an AUC of 0.89 and precision of 0.81, SVM showed an AUC of 0.93 and precision of 0.90, the random forest achieved an AUC of 0.95 and precision of 0.93, and XGBoost scored an AUC of 0.95 and precision of 0.92. In external validation, logistic regression achieved an AUC of 0.81 and precision of 0.79, SVM obtained an AUC of 0.87 and precision of 0.76, the random forest reached an AUC of 0.92 and precision of 0.86, and XGBoost recorded an AUC of 0.93 and precision of 0.91.

**Conclusion:**

The machine learning models performed better in predicting ICH recurrence than traditional statistical models. The XGBoost model demonstrated the best comprehensive performance for predicting ICH recurrence in the external testing cohort.

## Introduction

Patients with intracerebral hemorrhage (ICH) have higher rates of disability and mortality compared to those with ischemic stroke (1, 2). Factors such as recurrence can lead to worsening conditions and increased mortality in ICH patients (3). The recurrence rate in ICH patients is approximately 2–10% (3). Consequently, predicting the recurrence risk and implementing preventive or therapeutic measures for patients at higher risk of recurrence is a rational strategy to improve outcomes potentially (3, 4).

Currently, there are a few studies on predicting the recurrence of ICH. One predictive model, which included 38 patients with recurrent ICH, demonstrated an AUC of 0.802 (5). However, the limited number of outcome events could lead to the model’s performance instability. Additionally, the model lacked an external cohort for validation, which is crucial for assessing its generalizability. Other recurrence model had a similar condition. Therefore, a model that includes more outcome events and an external validation cohort is necessary to predict ICH recurrence more accurately.

Numerous risk factors can influence the recurrence rate (6). For example, the patient’s condition, lobar cerebral hemorrhages or deep subcortical intracerebral hemorrhages, amount of bleeding, changes in the amount of bleeding, and medication status after discharge should be documented (6). These factors, with their intricate correlations, also impact the accuracy of predictions (6). All these aspects affect the performance of traditional statistical models. Machine learning models could appropriately address these challenges (7, 8). Two studies constructed machine learning models to predict outcomes in ICH patients, outperforming traditional statistical models and scores (9, 10). One study developed a machine learning model to predict the occurrence of seizures in ICH patients, which also performed well (11).

In this study, we aim to construct a machine-learning model to predict the recurrence of ICH. The model incorporates a larger cohort of patients and more outcome events. Furthermore, we validated the model’s generalizability using an external cohort.

## Methods

### Study subjects

The training cohort of ICH patients was drawn from neurosurgery, neurology, and rehabilitation departments at the affiliated hospitals Youjiang Medical University for Nationalities. The inclusion period was from January 1, 2018, to January 1, 2021, with follow-up extending to January 1, 2022. This was a retrospective cohort. The testing cohort of ICH patients originated from the affiliated Baidong hospitals of Youjiang Medical University for Nationalities, from January 1, 2021, to January 1, 2022, and follow-up until January 1, 2023. This was a prospective cohort.

Inclusion criteria: 1. Aged 18 years or older who underwent head CT examinations and met the WHO’s diagnostic criteria for ICH. 2. Standard medical or surgical treatment was administered to all patients. 3. First ICH patients.

Exclusion criteria: 1. Traumatic cerebral hemorrhage, ICH due to venous sinus thrombosis, metastatic lesions, or underlying vascular lesions. 2. Recurrence of intracerebral hemorrhage secondary to infection or hemorrhagic cerebral infarction excluded. 3. The patient died during hospitalization.

The study was conducted by the Declaration of Helsinki and the ethical standards of the institutional and/or national research committee. The study received approval from the Ethics Committee of the Affiliated Hospital of Youjiang Medical University for Nationalities. Informed consent was obtained from all study participants or their surrogates. Patients who could not give consent and those without a legally appointed representative were excluded from our study.

### Risk factors and outcomes

Patient data were collected from electronic medical records, including demographic information such as age, gender, and nationality. Vital signs, including heart rate and blood pressure, were recorded at admission. Comorbidities, such as renal insufficiency, epilepsy, pneumonia, and the location of ICH, were also documented. Laboratory data, including PLT (blood platelet), INR (international normalized ratio), ALT (glutamic-pyruvic transaminase), AST (glutamic oxalacetic transaminase hemoglobin), LDL-C (low-density lipoprotein), CRP (C Reactive Protein), and others, were collected. Medical histories and medication profiles were obtained using structured questionnaires completed by patients or their relatives.

Clinical assessments, such as NIHSS (National Institute of Health stroke scale) scores at admission and discharge, mRS (Modified Rankin Scale) scores at admission and discharge, GCS (Glasgow Coma Scale) scores at admission, and ADL (activity of daily living) scores at admission, were conducted. Hematoma volumes were measured using the ABC/2 method and evaluated by the Alice software (PAREXEL International, Waltham, MA, United States) (12). Two experienced neurologists blinded to the patient’s conditions and outcomes performed these evaluations at admission and discharge.

The outcome was the recurrence of ICH within 1 year post-onset. We follow up with each patient until 1 year after the onset of the disease, and document any occurrences of recurrent intracerebral hemorrhage or death within the year. Head CT examinations confirmed recurrent cases according to the WHO’s ICH diagnostic criteria. The neurologists evaluated these data independently of the patient’s other conditions and baseline data.

The training and testing cohorts were subjected to the same evaluation and inclusion criteria, and the uniform process collected data for both cohorts. Both cohorts shared similar baseline characteristics. All laboratory data adhered to a uniform standard for normal values.

### Availability of data and material

Data from the study are available from the corresponding author upon.

### Statistical analysis and machine learning model

After gathering and processing data from all patients, we performed statistical and machine learning modeling.

The number of missing features constituted less than one-fifth of the total variables in the cohort. We used multiple imputations to address missing data. Continuous variables were not converted into categorical variables. All data from the final cohort were included in the model. Statistical analyses were performed using SPSS 23.0 for Windows and Python 3.8.0. The threshold for statistical significance was set at *p* < 0.05.

#### Baseline characteristics between cohorts

We presented normally distributed continuous variables as mean ± SD (standard deviation) and non-normally distributed as median and frequencies. These data were compared using a t-test for normally distributed variables (such as blood pressure, heart rate, laboratory data, etc.) or a Mann–Whitney U test for non-normally distributed variables (such as NIHSS, ADL, GCS, etc.) between the two cohorts. Categorical variables (such as gender, history of disease, history of medication, etc.) or ranked variables (such as mRS, occupation, nationality, etc.) were expressed as numbers and percentages. These data were compared using the chi-square test.

#### Data pre-processing

To mitigate the significant impact of class imbalance on machine learning performance, we applied random under-sampling (RUS) technique to balance the data (Scikit-learn library in Python). To guarantee fairness in the under-sampling process and unbiased generalization capabilities of the model, we performed 200 repeated experiments with distinct random under-sampling in each, coupled with ten-fold cross-validation to examine the results’ stability.

#### Feature selection

Our data included 70 variables. We conducted feature selection using various methods to identify relevant variables for the model. We selected relevant features through Lasso regression and a step-by-step recursive procedure (Boruta library in Python). When the *p*-value of the feature is less than 0.05, the feature is included in the model. Additional features were identified and included based on clinical guidelines and literature (4–6).

#### Construction and internal validation of the model

We choose commonly used and well-performing basic machine learning algorithms (logistic regression, support vector machine, and decision trees) and integrated machine learning algorithms (Voting Classifier model, random forest model, and XGBoost model) that have shown good performance in previous medical research to build the model (7–11).

We constructed three fundamental machine learning algorithms: logistic regression, support vector machine (SVM), and decision trees (sklearn library in Python). We employed a five-fold cross-validation (4:1) for randomly splitting the training cohort’s data. Subsequently, we performed grid search and cross-validation to optimize parameters (sklearn library in Python). We also calculated the ROC and precision values and depicted the model performance using ROC and precision plots (sklearn library in Python).

We further constructed integrated algorithm models based on three fundamental algorithms. The integrated algorithm included a Voting Classifier model, random forest model, and XGBoost model (sklearn library in Python). We calculated the F1 score for each integrated algorithm model and comprehensively compared the performance of each model through AUC, precision values, and F1 scores. We evaluated whether these integrated algorithms improved the performance of the fundamental algorithm models and selected the model with the best performance.

#### External validation of the model

We addressed outliers and class imbalance in the testing cohort data similarly to the training cohort data. We then predicted the recurrence of ICH in the testing cohort data (without outcome events) using the selected model from the previous step. The actual outcome events were used to validate the model’s performance. We demonstrated the model’s external performance and generalization ability through AUC and prediction accuracy.

#### Model visualization

To further elucidate the selected model, we visualized the model using feature importance and individual prediction. We depicted the importance of each feature in the final model with bar charts (SHAP library in Python). We illustrated the effect of each feature on individual patient predictions using visual representations (LIME library in Python).

## Results

### Baseline characteristics

Initially, 1,133 patients were eligible for the training cohort; however, we excluded 129 patients due to loss of follow-up, missing data, or withdrawal from the study. In the end, the training cohort included 1,024 patients, with 114 experiencing ICH recurrence, and 203 patients who passed away (176 due to neurological causes and 27 due to non-neurological causes). The training cohort had 53 patients with missing data, which we addressed through multiple imputations. The mean age of the training cohort was 63.50 ± 13.550 years, 43.3% of whom were female (443 patients). After excluding 24 patients for the same reasons as in the training cohort, the test cohort comprised 265 patients, with 31 experiencing ICH recurrence, and 41 patients who passed away (36 due to neurological causes and 5 due to non-neurological causes). Similarly, 11 patients in the test cohort had missing data, which we addressed through multiple imputations. The mean age of the test cohort was 59.82 ± 13.634 years, 40.4% of whom were female (107 patients) (Supplementary Figure S1).

In the training cohort, patients with recurrence were less likely to take hypotensive drugs post-onset and had less well-controlled blood pressure. Additionally, patients with recurrence were older and had greater hematoma volume at admission and discharge, higher NIHSS and ADL scores, and higher PLT levels at admission. Other features showed no significant differences (Table 1).

**Table 1:** Baseline characteristic of training cohort.

Feature	All patients (*N* = 1,024)	Recurrence (*N* = 114)	No-Recurrence (*N* = 910)	p*
Age, years	63.50 (13.550)	68.81 (12.960)	62.84 (13.483)	<0.001
Female, %	443 (43.3)	48 (42.1)	395 (43.4)	0.361
Discharge NIHSS score	6 (1–15)	20 (15–35)	4 (1–11)	<0.001
Admission ADL score	40 (20–70)	25 (10–45)	45 (25–75)	<0.001
Admission hematoma, mL	21 (11–24)	25 (22–28)	19 (10–24)	<0.001
Discharge hematoma, mL	13 (7–22)	36 (32–40)	12 (6–19)	<0.001
History of stroke, %	277 (27.1)	26 (22.8)	251 (27.6)	0.137
Hypotensive drugs, %	826 (80.7)	67 (58.8)	759 (83.4)	0.003
Platelet, mmol/L	202.80 (71.176)	179.78 (64.950)	205.69 (71.429)	<0.001
AST, mmol/L	26.48 (16.340)	26.57 (17.264)	26.47 (16.230)	0.949
CRP, mmol/L	19.55 (16.975)	20.88 (17.470)	19.39 (17.155)	0.375

p* was calculated by ANOVA, Chi-square test, or Mann–Whitney U test as appropriate. NIHSS, National Institute of Health stroke scale; ADL, activity of daily living; AST, glutamic oxalacetic transaminase; CRP, C-reactive protein.

In the test cohort, patients with recurrence had higher NIHSS and ADL scores at discharge and greater hematoma volume at admission and discharge. Other features showed no significant differences (Table 2). Both cohorts had a similar distribution in baseline characteristics.

**Table 2:** Baseline characteristic of testing cohort.

Feature	All patients (*N* = 265)	Recurrence (*N* = 31)	No-Recurrence (*N* = 234)	p*
Age, years	59.82 (13.634)	61.48 (12.951)	59.60 (13.733)	0.471
Female, %	107 (40.4)	13 (41.9)	94 (40.2)	0.615
Discharge NIHSS score	5 (1–17)	18 (11–35)	4 (1–12)	<0.001
Admission ADL score	50 (0–85)	45 (10–65)	65 (15–90)	<0.001
Admission hematoma, mL	18 (11–25)	28 (26–29)	16 (10–23)	<0.001
Discharge hematoma, mL	12 (6–25)	30 (27–32)	10 (5–20)	<0.001
History of stroke, %	56 (21.1)	6 (19.4)	50 (21.4)	0.657
Hypotensive drugs, %	228 (86.0)	24 (77.4)	204 (87.2)	0.198
Platelet, mmol/L	200.39 (70.061)	193.45 (52.069)	202.19 (72.113)	0.444
AST, mmol/L	26.77 (15.174)	27.32 (18.707)	26.70 (14.689)	0.831
CRP, mmol/L	19.67 (14.017)	19.93 (13.336)	19.62 (14.132)	0.910

p* was calculated by ANOVA, Chi-square test, or Mann–Whitney U test as appropriate. NIHSS, National Institute of Health stroke scale; ADL, activity of daily living; AST, glutamic oxalacetic transaminase; CRP, C-reactive protein.

To compare the baseline data between the training and test cohorts, patients in the training cohort were older than those in the test cohort. Other baseline data showed no significant difference between the two cohorts (Table 3).

**Table 3:** Baseline characteristic between training and testing cohort.

Feature	Training set (*N* = 1,024)	Testing set (*N* = 265)	p*
Age, years	63.50 (13.550)	59.82 (13.634)	<0.001
Female, %	443 (43.3)	107 (40.4)	0.317
Discharge NIHSS score	6 (1–15)	5 (1–17)	0.279
Admission ADL score	40 (20–70)	50 (0–85)	0.273
Admission hematoma, mL	21 (11–24)	18 (11–25)	0.116
Discharge hematoma, mL	13 (7–22)	12 (6–25)	0.907
History of stroke, %	277 (27.1)	56 (21.1)	0.317
Hypotensive drugs, %	826 (80.7)	228 (86.0)	0.203
Platelet, mmol/L	202.80 (71.176)	200.39 (70.061)	0.622
AST, mmol/L	26.48 (16.340)	26.77 (15.174)	0.790
CRP, mmol/L	19.55 (16.975)	19.67 (14.017)	0.917

p* was calculated by ANOVA, Chi-square test, or Mann–Whitney U test as appropriate. NIHSS, National Institute of Health stroke scale; ADL, activity of daily living; AST, glutamic oxalacetic transaminase; CRP, C-reactive protein.

### Data pre-processing and feature selection

Through random under-sampling and cross-validation, the model performance has improved by approximately 10%.

Through Lasso regression and a stepwise process, we identified several relevant variables for the model, including age, NIHSS score at discharge, hematoma volume at admission and discharge, and PLT, AST, and CRP levels at admission. Subsequently, based on clinical significance and literature (4–6), and for enhanced model performance after adding additional variables, particularly the improvement in model performance during external validation, we have chosen to incorporate the use of hypotensive drugs and history of stroke into the model.

### Internal validation performance of models

For the basic machine learning models, internal validation revealed that the AUC for the logistic regression model was 0.89, and the precision was 0.81, the positive predictive value (PPV) was 87.3%, and negative predictive value (NPV) was 76.0%. The SVM model had an AUC of 0.93 and a precision of 0.90, a PPV of 94.2% and a NPV of 88.5%, while the decision trees model showed an AUC of 0.91 and a precision of 0.71, a PPV of 79.0% and a NPV of 81.5%. All three models demonstrated excellent discrimination, with the logistic regression and SVM models also exhibiting excellent calibration.

For the integrated machine learning models, the score for the Logistic Regression model was 0.951, for the Decision Tree Classifier was 0.962, and for the SVC model was 0.883. In the Voting Classifier model, the score for the soft voting method was 0.928, with an AUC and precision of 0.96 and 0.86, a PPV of 94.2% and a NPV of 89.4%, respectively. The random forest model scored 0.966 and exhibited an AUC and precision of 0.95 and 0.93, a PPV of 95.0% and a NPV of 92.3%, respectively. The XGBoost model scored 0.977, with an AUC and precision of 0.95 and 0.92, a PPV of 94.6% and a NPV of 93.1%, respectively. All three integrated machine learning models exhibited excellent discrimination, and the latter two models demonstrated excellent calibration. The random forest and XGBoost algorithms improved the basic models’ performance.

### External validation performance of models

External validation for the basic machine learning models showed that the AUC for the logistic regression model was 0.81 (Supplementary Figure S3a-1), with a precision of 0.79 (Supplementary Figure S3a-2). The SVM model had an AUC of 0.87 (Supplementary Figure S3b-1) and a precision of 0.76 (Supplementary Figure S3b-2), and the decision trees model had an AUC of 0.71 (Supplementary Figure S3c-1) and a precision of 0.70 (Supplementary Figure S3c-2).

For the integrated machine learning models, the random forest model exhibited an AUC of 0.92 (Supplementary Figure S4a-1) and a precision of 0.86 (Supplementary Figure S4a-2), while the XGBoost model had an AUC of 0.93 (Supplementary Figure S4b-2) and a precision of 0.91 (Supplementary Figure S4b-2). The random forest model had a PPV of 90.0% and a NPV of 85.2%, and the XGBoost model had a PPV of 92.5% and a NPV of 93.2%. The integrated machine learning algorithms still demonstrated excellent discrimination and calibration in the test cohort.

### Model visualization

**Figure 1:**
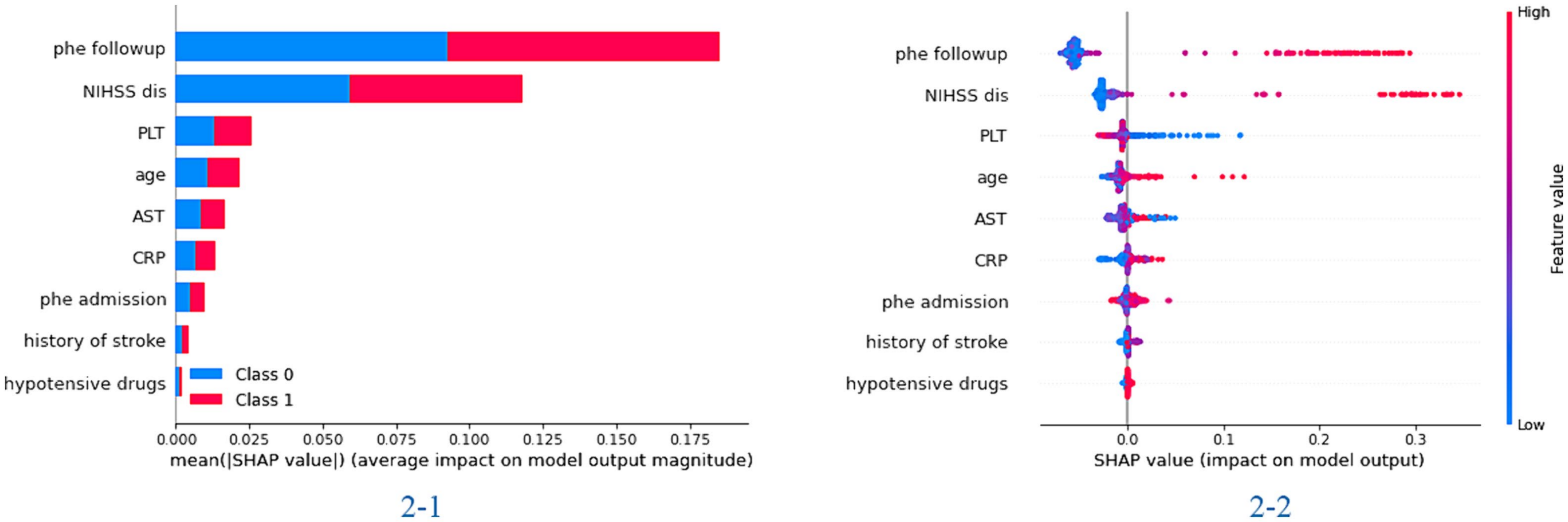
The importance of the feature plot of the XGBoost model. 2-1: the importance of features for predicting in the total model; 2-2: the importance of features for predicting in each patient of the model.

In the feature importance figure of the XGBoost model, we found that the value of the hematoma and NIHSS scores at discharge had the most significant effect on the model. Then, the age, the value of PLT and AST, and the value of hematoma at admission also affected the model differently (Figure 1). In the feature importance figure of the random forest model, we found that the value of hematoma at discharge had the best significant effect on the model. Then, the NIHSS score at discharge, the value of hematoma at admission, age, and the value of PLT and AST also affected the model differently (Supplementary Figure S2).

**Figure 2:**
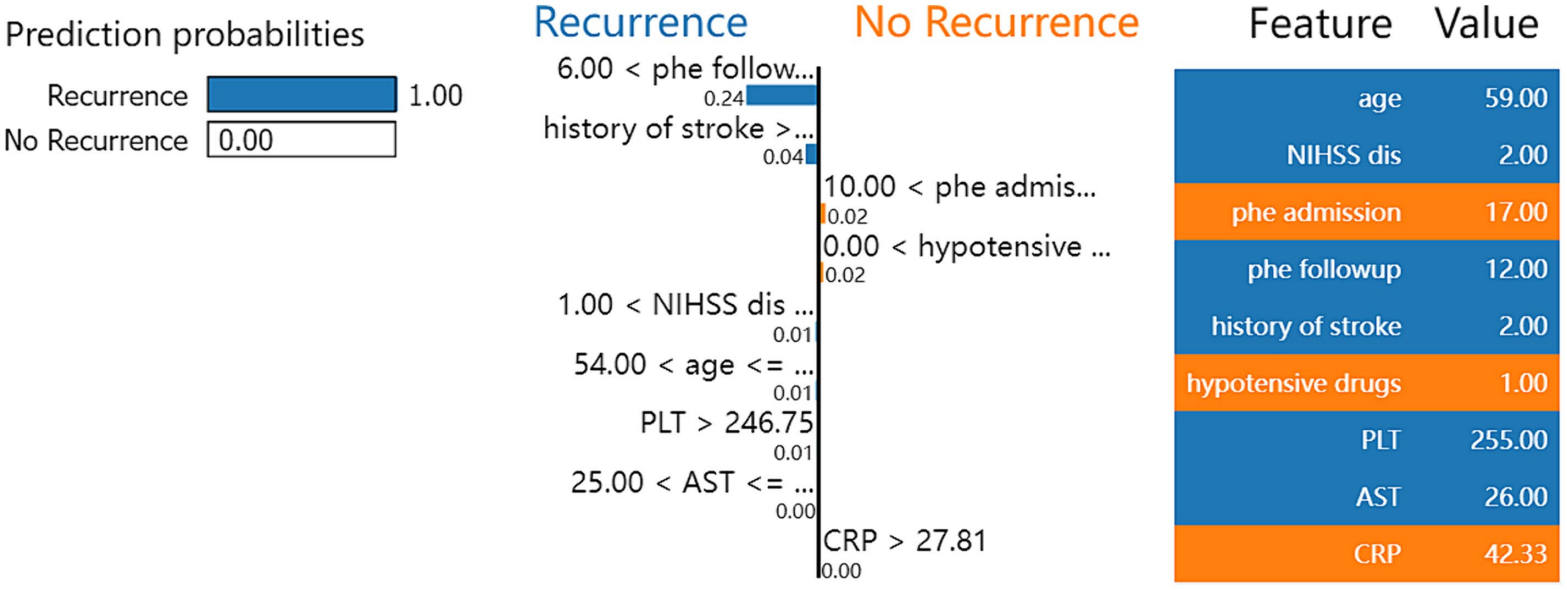
Local interpretable model-agnostic explanation (LIME) with XGBoost model applied to one correctly predicted patient that ICH recurrence within 1 year.

Figure 2 shows the process of the XGBoost model to predict the recurrence rate in individual ICH patients. First, the XGBoost model calculated the score through the difference in volume extent of hematoma and NIHSS score, age, PLT, and AST. Then, the model calculated the possibility of ICH recurrence in the individual patients. For the selected patients, the model accurately predicts the patients would have recurrent ICH with a 100% possibility.

## Discussion

The study constructs machine learning models to predict the recurrence of ICH. We evaluated six machine learning models. The models included age, NIHSS score at discharge, and hematoma volume at admission and discharge. The SVM and decision trees were performed better than logistic regression model in the training cohort. The integrated machine learning models performed better than the basic models in the training cohort. In the test cohort, the AUC and precision of the basic models all decreased to less than 0.9. However, the integrated models, such as the random forest and XGBoost models, still performed better. Notably, the XGBoost model performed excellently in the testing cohort, with an AUC and precision greater than 0.9.

We performed multiple imputation on data with a small number of missing features, and data with more than 30% missing features were not included in the analysis. Less than 1% of the data was not included in the analysis, and it did not significantly affect the result analysis. We standardized all the data included in our model; thus, the data distribution did not significantly affect the results.

For selected features, the NIHSS score at discharge had a highly significant impact on the model. Their importance was ranked second and third in the final random forest and XGBoost models. The neurological deficit at discharge contained more information for predicting long-term events. The discharge score at least reflects the illness’s initial state and therapy’s effect. In other study, the discharge score had a more pronounced relationship with patient outcomes (5, 9–11). The hematoma volume also had a similar effect. Our model included hematoma volume at admission and discharge, allowing it to evaluate the changing situation throughout hospitalization. These changes could more accurately reflect the patients’ conditions. This was similar to other models, including the GCS score at admission and discharge (5). The hematoma volume consistently ranked among the top three in our final random forest and XGBoost models, significantly improving our model’s performance.

Our model also included age and PLT levels. It is plausible that older age and lower PLT levels could increase the risk of ICH recurrence, which is consistent with other studies (13). Our model also indicated that AST and CRP levels affect the risk of recurrence. Poor liver function and infection can affect the state of the illness (14, 15). However, these effects require further investigation. Our model includes a history of stroke and the use of hypotensive drugs. Although these two features did not significantly affect our model performance, these clinically significant features could improve the model’s generalizability. Therefore, our model could perform better in different region cohorts. An acute intraparenchymal hematoma in a young patient can be the presenting manifestation of a hematological disorder (16). The hematologic disorders from other stroke etiologies that have a different treatment approach and outcome (16). For less young patients in our cohort, a model included more young patients was more suit for these patients with hematological disorder.

The recurrence of ICH can be influenced by numerous risk factors that vary over time. These factors exhibit a complex interrelationship that traditional statistical models struggle to represent. Machine learning models are more adept at processing these complex characteristics. Consequently, machine learning models have the potential to identify novel predictors with enhanced predictive value. They demonstrated excellent performance in the training cohort due to the abovementioned advantages. However, machine learning models face challenges when applied to external datasets. In other words, they may exhibit poorer generalization performance. The overall performance of our model decreased in the test cohort. Nevertheless, the integrated model exhibited superior generalization capabilities, maintaining its excellent performance even within the test cohort. Consequently, the integrated model could hold significant clinical value across various regions.

Another common challenge with machine learning models is the interpretability of their outcomes. The SHAP library is widely used for global interpretation of model outcomes, revealing the significance of each model feature (17). This means the library elucidates the impact and contribution of each feature to the predictive results and displays each feature’s value within the model. The LIME library, which stands for Local Interpretable Model-Agnostic Explanations, can demonstrate the model’s prediction process for individual samples (18). Consequently, LIME visually illustrates how the model predicts the likelihood of outcome events based on the value of each feature. These two libraries enhance our model’s interpretability, demonstrating practical value and superior performance in clinical settings.

This study faced several limitations. First, the training cohort might be susceptible to bias owing to its retrospective design. Second, the training and testing cohorts, which originated from the same geographical region, had a similar feature distribution. Consequently, it becomes imperative to validate the model’s performance across diverse regional patient populations further. Third, our model did not incorporate neuroimaging data; including a broader array of data types could potentially enhance model performance.

## Conclusion

The machine learning model demonstrated excellent performance in predicting the recurrence of ICH compared to traditional statistical models. The XGBoost models with hematoma volume at admission and discharge outperformed the external test cohort. The change situation in hematoma volume improves the performance of model. The XGBoost model exhibited the best discrimination and calibration in predicting the recurrence of ICH. The XGBoost model offered excellent predictive value for guiding medical care in patients at a higher risk of ICH recurrence.

The use of this XGBoost model, along with other machine learning models for predicting recurrent intracerebral hemorrhage, can improve the accuracy of recurrent intracerebral hemorrhage prediction. It guides the formulation of personalized treatment and prevention plans in clinical medicine. The next step involves validating the model’s applicability in other regions and ethnicities to confirm its practicality and universality.

## Data availability statement

The raw data supporting the conclusions of this article will be made available by the authors, without undue reservation.

## Ethics statement

The studies involving humans were approved by the Ethics Committee of the Affiliated Hospital of Youjiang Medical University for Nationalities. The studies were conducted in accordance with the local legislation and institutional requirements. The participants provided their written informed consent to participate in this study.

## Author contributions

CC: Conceptualization, Formal analysis, Funding acquisition, Methodology, Software, Supervision, Writing – original draft, Writing – review & editing. JL: Conceptualization, Formal analysis, Writing – review & editing. ZL: Data curation, Investigation, Methodology, Software, Writing – review & editing. TX: Data curation, Investigation, Methodology, Writing – review & editing. TL: Data curation, Investigation, Software, Writing – review & editing.

## References

[1] Hemphill JC Greenberg SM Anderson CS Becker K Bendok BR Cushman M. Guidelines for the management of spontaneous intracerebral hemorrhage. Stroke. (2015) 46:2032–60. doi: 10.1161/str.0000000000000069 26022637

[2] Geurts M Macleod MR van Thiel G van Gijn J Kappelle LJ van der Worp HB. End-of-life decisions in patients with severe acute brain injury. Lancet Neurol. (2014) 13:515–24. doi: 10.1016/s1474-4422(14)70030-4 24675048

[3] Wolf ME Alonso A Ebert AD Szabo K Chatzikonstantinou A. Etiologic and clinical characterization of patients with recurrent spontaneous intracerebral hemorrhage. Eur Neurol. (2016) 76:295–301. doi: 10.1159/000452659, PMID: 27806359

[4] Huhtakangas J Löppönen P Tetri S Juvela S Saloheimo P Bode MK. Predictors for recurrent primary intracerebral hemorrhage a retrospective population-based study. Stroke. (2013) 44:585–90. doi: 10.1161/strokeaha.112.671230, PMID: 23329207

[5] Zhang S Zhang X Ling Y Li A. Predicting recurrent hypertensive intracerebral hemorrhage: derivation and validation of a risk-scoring model based on clinical characteristics. World Neurosurg. (2019) 127:E162–71. doi: 10.1016/j.wneu.2019.03.024, PMID: 30876994

[6] de Mendiola J Arboix A García-Eroles L Sánchez-López MJ. Acute spontaneous lobar cerebral hemorrhages present a different clinical profile and a more severe early prognosis than deep subcortical intracerebral hemorrhages-a hospital-based stroke registry study. Biomedicines. (2023) 11:11. doi: 10.3390/biomedicines11010223, PMID: 36672731 PMC9856131

[7] Cui CH Li YC Liu SH Wang P Huang Z. The unsupervised machine learning to analyze the use strategy of statins for ischaemic stroke patients with elevated transaminase. Clin Neurol Neurosurg. (2023) 232:107900. doi: 10.1016/j.clineuro.2023.107900, PMID: 37478641

[8] Cui CH Li CH Hou M Wang P Huang Z. The machine learning methods to analyze the using strategy of antiplatelet drugs in ischaemic stroke patients with gastrointestinal haemorrhage. BMC Neurol. (2023) 23:369. doi: 10.1186/s12883-023-03422-0, PMID: 37833629 PMC10571309

[9] Wang HL Hsu WY Lee MH Weng HH Chang SW Yang JT. Automatic machine-learning-based outcome prediction in patients with primary intracerebral hemorrhage. Front Neurol. (2019) 10:7. doi: 10.3389/fneur.2019.00910, PMID: 31496988 PMC6713018

[10] Hall AN Weaver B Liotta E Maas MB Faigle R Mroczek DK. Identifying modifiable predictors of patient outcomes after intracerebral hemorrhage with machine learning. Neurocrit Care. (2021) 34:73–84. doi: 10.1007/s12028-020-00982-8, PMID: 32385834 PMC7648730

[11] Bunney G Murphy J Colton K Wang H Shin HJ Faigle R. Predicting early seizures after intracerebral hemorrhage with machine learning. Neurocrit Care. (2022) 37:322–7. doi: 10.1007/s12028-022-01470-x, PMID: 35288860 PMC10084721

[12] Kothari U Brott T Broderick JP Barsan WG Sauerbeck LR Zuccarello M. The ABCs of measuring intracerebral hemorrhage volumes. Stroke. (1996) 27:1304–5. doi: 10.1161/01.Str.27.8.1304, PMID: 8711791

[13] Naidech AM Bendok BR Garg RK Bernstein RA Alberts MJ Bleck TP. Reduced platelet activity is associated with more intraventricular hemorrhage. Neurology. (2009) 72:A73–3. doi: 10.1227/01.NEU.0000351769.39990.16 19834372

[14] Ruban A Daya N Schneider ALC Gottesman R Selvin E Coresh J. Liver enzymes and risk of stroke: the atherosclerosis risk in communities (ARIC) study. J Stroke. (2020) 22:357. doi: 10.5853/jos.2020.00290, PMID: 33053951 PMC7568972

[15] Diedler J Sykora M Hahn P Rupp A Rocco A Herweh C. C-reactive-protein levels associated with infection predict short- and long-term outcome after Supratentorial intracerebral hemorrhage. Cerebrovasc Dis. (2009) 27:272–9. doi: 10.1159/000199465, PMID: 19202332

[16] Arboix A Besses C. Cerebrovascular disease as the initial clinical presentation of haematological disorders. Eur Neurol. (2004) 37:207–11. doi: 10.1159/000117444 9208259

[17] Wang K Tian J Zheng C Yang H Ren J Liu Y. Interpretable prediction of 3-year all-cause mortality in patients with heart failure caused by coronary heart disease based on machine learning and SHAP. Comput Biol Med. (2021) 137:9. doi: 10.1016/j.compbiomed.2021.104813, PMID: 34481185

[18] Hussain I Jany R Boyer R Azad AKM Alyami SA Park SJ. An explainable EEG-based human activity recognition model using machine-learning approach and LIME. Sensors. (2023) 23:15. doi: 10.3390/s23177452, PMID: 37687908 PMC10490625

