# Supplementary material for: Predicting the recurrence of spontaneous intracerebral hemorrhage using a machine learning model
Source: Front Neurol. 2024 May 22;15:1407014. doi: 10.3389/fneur.2024.1407014 (PMC11150637; doi:10.3389/fneur.2024.1407014)
Supplement: Supplementary file 1 [file Data_Sheet_1.docx]

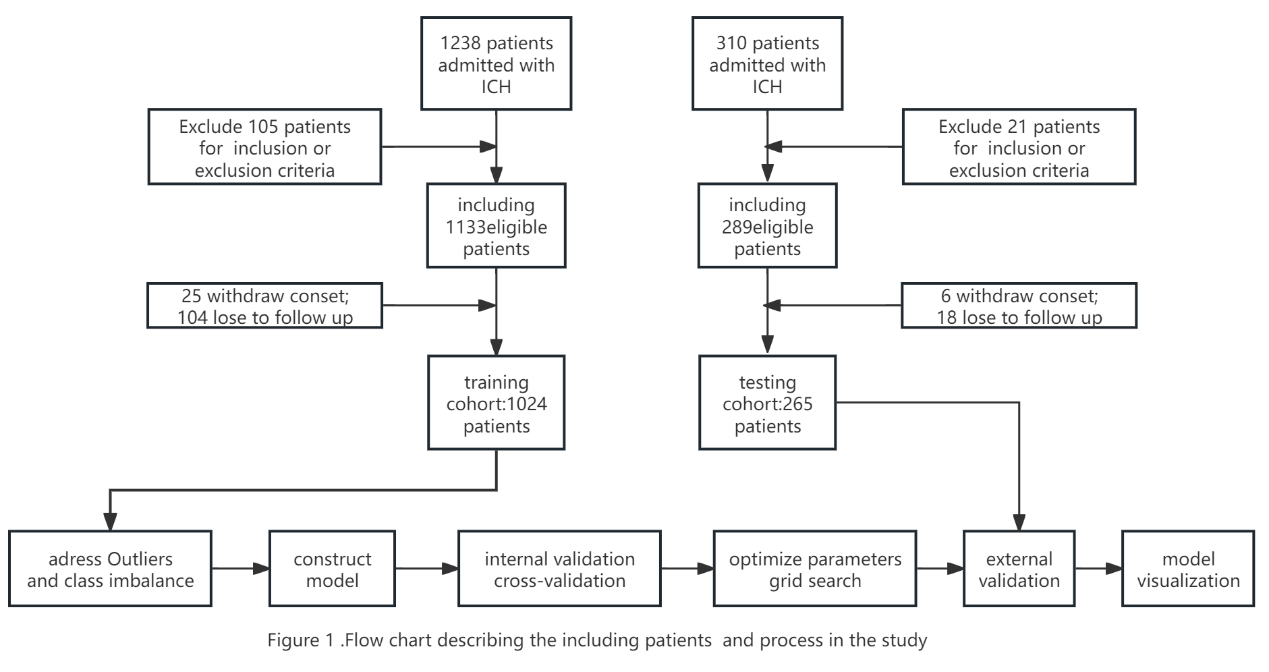


Supplementary figure 01: the flow chart including patients and study process


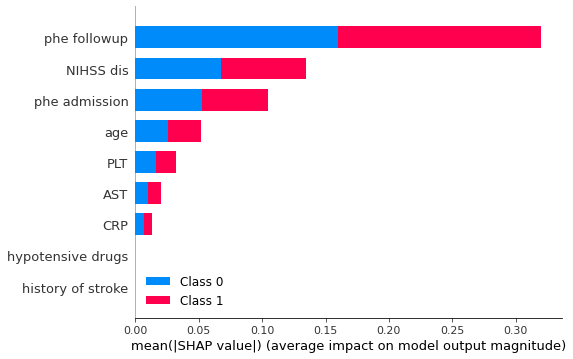


Supplementary figure 02: The importance of feature plot of random forest model.


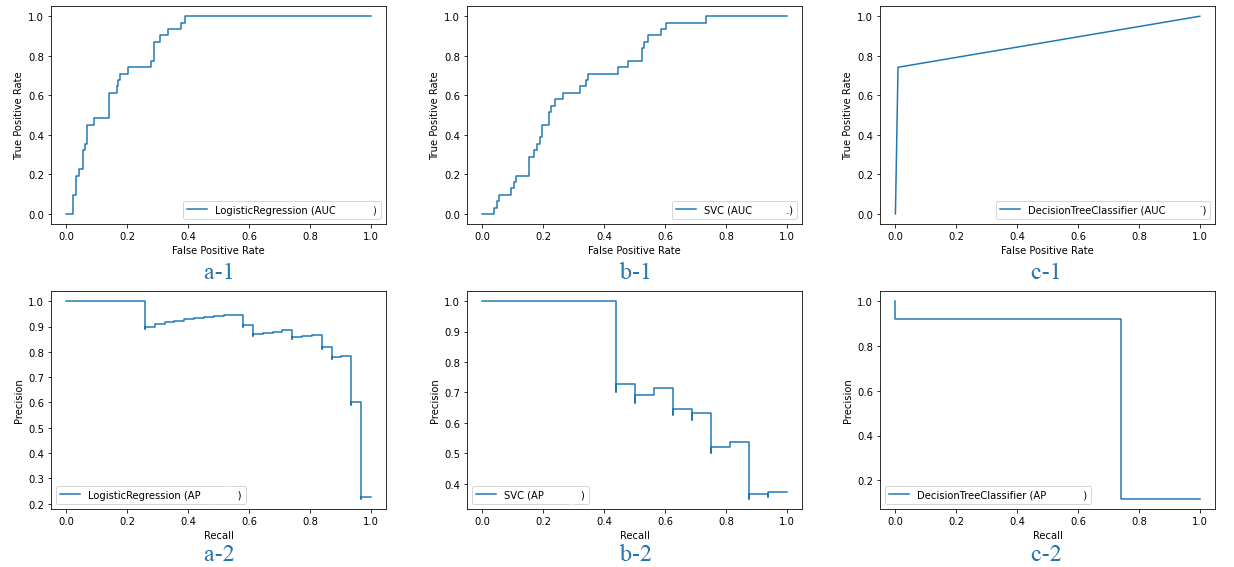


Supplementary figure 03: The ROC and Precision plot of the basic model for external validation. a-1: ROC of logistic regression model; a-2 Precision of logistic regression model; b-1 ROC of support vector machine model; b-2 Precision of support vector machine model; c-1 ROC of decision trees model; c-2 Precision of decision trees model.


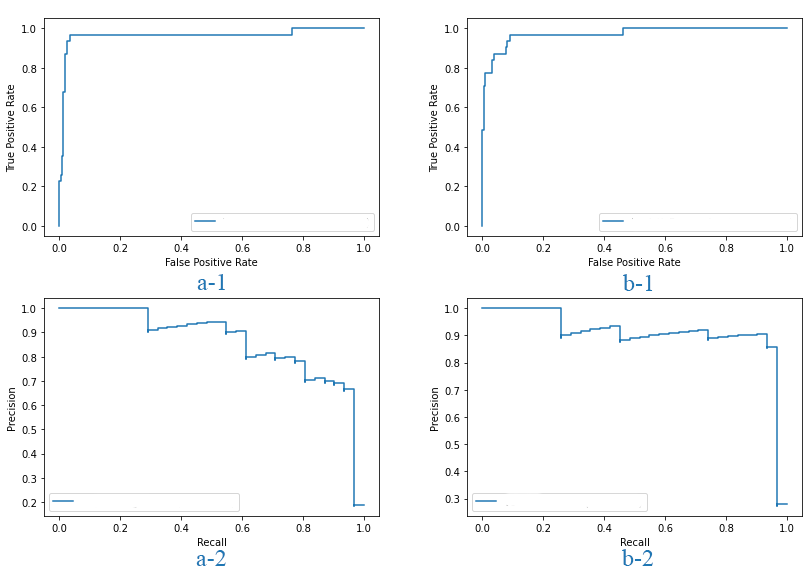


Supplementary figure 04: The ROC and Precision plot of the integrated model for external validation. a-1: ROC of random forest model; a-2 Precision of random forest model; b-1 ROC of XGBoost model; b-2 Precision of XGBoost model.
